# Effect of CYP2C9 Polymorphism on Neuropsychiatric Symptoms in Alzheimer’s Dementia

**DOI:** 10.1002/alz.095525

**Published:** 2025-01-09

**Authors:** Evelyn Gutiérrez, Maria Alejandra Tangarife, Jagadeesh S Rao, Margarita Venegas, María Juanita Arbeláez, Laura Tatiana Sánchez, William Julio De Jesus, Karen Y Pujals, Claudia Grimaldi, Elliot Hong, Ram Mukunda

**Affiliations:** ^1^ IGC Pharma, Bogotá, Bogotá D.C. Colombia; ^2^ IGC Pharma, Potomac, MD USA; ^3^ Santa Cruz Behavioral, Bayamon, PR USA; ^4^ UTHealth Houston McGovern Medical School, Houston, TX USA

## Abstract

**Background:**

Alzheimer’s Disease (“AD”) presents a significant global health burden, often requiring medication management of comorbidities, some of which are metabolized by the polymorphic enzyme CYP2C9. We investigated the impact of CYP2C9 polymorphism on the reduction of Neuropsychiatric Inventory (NPI‐12) scores following administration of IGC‐AD1, comprising THC and melatonin, in AD patients.

**Method:**

Thirteen Puerto Rican AD patients (mean age: 80.18±6.22 years, 70% women) participated in a Phase‐1 trial. Ten active participants received IGC‐AD1 once a day (“Cohort 1”, “QD”), twice a day (“Cohort 2”, “BID”), or thrice daily (“Cohort 3”, “TID”) for 14 days (“EOT”) with a washout period. NPI‐12 was assessed at baseline and endpoint, and participants were grouped by CYP2C9 phenotype. CYP2C9 polymorphisms include normal (“NM”, allele *1/*1), intermediate (“IM”, alleles *1/*2, *1/*3), and poor (“PM”, alleles *2/*2, *3/*3, and *2/*3) metabolizers, among others. Wilcoxon signed‐rank and t‐tests compared baseline to endpoint NPI‐12 scores.

**Result:**

No significant associations were found between CYP2C9 NM and IM phenotypes in reducing NPI‐12 scores from baseline to EOT across all cohorts. However, NM showed higher NPI reductions than IM in Cohorts 1 (NM: 53.95%, IM: 45.65%) and 2 (NM: 67.65%, IM: 32.64%). In Cohort 3, IM had a comparable reduction (NM: 33.33%, IM: 35.04%).

**Conclusion:**

IGC‐AD1 demonstrated tolerability and potential efficacy in reducing Neuropsychiatric Symptoms in AD, with NM showing greater reductions after 1‐ and 2‐mL administrations, though similar reductions were observed with 3 mL. Further investigation with a larger sample is continuing.

